# Cultivated Vaginal Microbiomes Alter HIV-1 Infection and Antiretroviral Efficacy in Colonized Epithelial Multilayer Cultures

**DOI:** 10.1371/journal.pone.0093419

**Published:** 2014-03-27

**Authors:** Richard B. Pyles, Kathleen L. Vincent, Marc M. Baum, Barry Elsom, Aaron L. Miller, Carrie Maxwell, Tonyia D. Eaves-Pyles, Guangyu Li, Vsevolod L. Popov, Rebecca J. Nusbaum, Monique R. Ferguson

**Affiliations:** 1 Department of Pediatrics, University of Texas Medical Branch, Galveston, Texas, United States of America; 2 Department of Microbiology and Immunology, University of Texas Medical Branch, Galveston, Texas, United States of America; 3 Department of Obstetrics and Gynecology, University of Texas Medical Branch, Galveston, Texas, United States of America; 4 Oak Crest Institute of Science, Pasadena, California, United States of America; 5 East Tennessee State University, Department of Internal Medicine, Division of Infectious Diseases, Johnson City, Tennessee, United States of America; 6 Department of Pathology, University of Texas Medical Branch, Galveston, Texas, United States of America; 7 Human Pathophysiology and Translational Medicine graduate program, University of Texas Medical Branch, Galveston, Texas, United States of America; 8 Department of Internal Medicine, Division of Infectious Diseases, University of Texas Medical Branch, Galveston, Texas, United States of America; Harvard Medical School, United States of America

## Abstract

There is a pressing need for modeling of the symbiotic and at times dysbiotic relationship established between bacterial microbiomes and human mucosal surfaces. In particular clinical studies have indicated that the complex vaginal microbiome (VMB) contributes to the protection against sexually-transmitted pathogens including the life-threatening human immunodeficiency virus (HIV-1). The human microbiome project has substantially increased our understanding of the complex bacterial communities in the vagina however, as is the case for most microbiomes, very few of the community member species have been successfully cultivated in the laboratory limiting the types of studies that can be completed. A genetically controlled ex vivo model system is critically needed to study the complex interactions and associated molecular dialog. We present the first vaginal mucosal culture model that supports colonization by both healthy and dysbiotic VMB from vaginal swabs collected from routine gynecological patients. The immortalized vaginal epithelial cells used in the model and VMB cryopreservation methods provide the opportunity to reproducibly create replicates for lab-based evaluations of this important mucosal/bacterial community interface. The culture system also contains HIV-1 susceptible cells allowing us to study the impact of representative microbiomes on replication. Our results show that our culture system supports stable and reproducible colonization by VMB representing distinct community state types and that the selected representatives have significantly different effects on the replication of HIV-1. Further, we show the utility of the system to predict unwanted alterations in efficacy or bacterial community profiles following topical application of a front line antiretroviral.

## Introduction

The vaginal mucosa is composed of a stratified squamous epithelium covered by a mucous and glycogen rich layer that supports colonization by highly complex and remarkably diverse bacterial communities. The symbiotic relationship between the bacterial community fed by the carbon sources provided by the vaginal epithelial cells (VEC) creates both physical and chemical barriers that serve as part of the front line defenses against infection. Doederlein and colleagues are credited with the first report of cultivation of vaginal bacteria in 1892 exclusively isolating Lactobacilli concluding that they were important contributors to vaginal health [1], [2]. The vaginal microbiome (VMB) is now known to be composed of many different species of bacteria that collaborate to enhance the health of the vaginal mucosa. Conversely, a common dysbiotic state, referred to as bacterial vaginosis (BV), occurs when Lactobacillus-dominated VMBs are displaced by pathogenic bacterial communities with inflammatory properties. BV is the most common cause of vaginal concern in adult women worldwide and represents roughly 22 million annual cases in the U.S. substantially adding to health care costs [3], [4]. Prevalence varies geographically and racially with rates as high as 1 in 2 women [5]. Clinically BV can be asymptomatic or include symptoms ranging from a malodorous discharge and elevated vaginal pH to inflammation and, in severe cases, fertility issues including pre-term labor and delivery [6]. Although still debated, a number of studies suggest there is a sexual transmission or enhancement component to BV acquisition [7]–[9] however the mechanisms leading to BV development are poorly defined. Established risk factors for BV include multiple sexual partners [9], lack of male circumcision [10], hygiene (e.g. douching) [11], [12], host genetic polymorphisms [13] and others. Current antibiotic treatments are disappointing with a high frequency of recurrence [5] likely due to the formation of resistant biofilms that provide effective survival niches [8]. BV biofilms have also been connected to transmission as well as altering the success of antimicrobial therapies to treat other sexually transmitted infections (STI) [8].

In addition to impacting antimicrobial therapy, clinical associations have established that BV VMBs increase susceptibility to infection by HIV-1 and other STI [14]–[16]. In HIV-infected women, BV VMBs also associate with elevated HIV-1 titers in vaginal fluids increasing the likelihood of transmission to naïve partners [17]. Global estimates suggest there are more than 34 million people infected with HIV-1 with up to 7,000 new infections each day and almost 2 million AIDS-related deaths annually [18], [19]. Because women are more susceptible to HIV infection than men, studies of the impact of specific species or bacterial partnerships in healthy and dysbiotic VMB are desperately needed. The majority of VMB bacteria have proven impossible to cultivate in the lab and to date no in vitro or animal model system has been reported that supports colonization by intact healthy or BV VMB communities. The lack of such models has severely impacted the ability to predict toxic effects of vaginal applicants on an otherwise protective VMB.

Recent clinical reports have established that vaginal applicants can alter VMB composition [20]. These studies have been completed using cultivation-independent molecular approaches that have substantially advanced our understanding of the composition of bacterial communities leading to solidly established associations between Lactobacilli-dominated VMB and protection against HIV-1 and other STI [14], [15], [21]. Vaginal applicants, including over the counter feminine hygiene products commonly used to address discharge, malodor and a variety of other concerns, are delivered by several modalities including gels, suppositories and intravaginal rings (IVR) that likely have differential impacts upon VMBs. These delivery methods have been utilized for a variety of prophylactic compounds collectively referred to as microbicides designed to provide protection against HIV-1 [22]. It is well reported that several lead microbicides, selected by standard efficacy testing paradigms, failed to protect treated women from acquiring HIV-1 or other STI [23], [24]. Subsequent clinical evaluations suggest that these failures may be caused by VMB disruption leading to inflamed or unhealthy mucosae that were actually predisposed to infection [20], [25]–[27]. Such failures have been blamed, in part, on inadequate efficacy and safety testing [28]–[30].

Cultivation-independent clinical sampling approaches including vaginal swabs, tissue biopsies and IVR evaluations have provided insights into VMB composition. These approaches have highlighted the dynamic and kinetically fluid interactions among VMB community members and with the VEC but are complicated by the genetic and environmental diversity influences that confound interpretation of these massive data sets. Specifically, next generation sequencing studies completed by several groups have solidly established the complexity and dynamic nature of VMB communities as well as the impact of race, menstruation, sampling method, etc. [31]–[35]. Using sequence data from a remarkably large cohort of women, Ravel and colleagues have suggested a categorization scheme based on the four Lactobacillus species that most frequently dominate healthy VMB [34]. Their system designates VMB community state types (CST) based on the predominance of one Lactobacillus species; *L. crispatus* (CST I), *L. gasseri* (CST II), *L. iners* (CST III) and *L. jensenii* (CST V). VMB with multiple Lactobacillus species rather than a single predominant organism are categorized as CST IVA. VMB that lack Lactobacilli and are commonly designated as BV are categorized as CST IVB.

Using this categorization system and a PCR-based approach to characterize cryopreserved VMB collected from women undergoing routine gynecological exams, we have extended our recently reported VEC stratified multilayer culture system [36] to show reliable colonization by intact VMB representing 5 of the 6 CST; a CST V VMB that met our inclusion criteria was not available in our current repository preventing its inclusion in these studies. The cultured VEC multilayers recapitulate key aspects of the vaginal mucosa including production of the necessary carbon sources and molecular signals to support the bacterial communities. This culture model supported the formation of VMB-associated biofilms that, in turn, altered the culture phenotype with regard to HIV-1 infection and topical antiretroviral therapy. For study of HIV-1 infection, the multilayer cultures were refined to include HIV-1 susceptible cells that allowed a controlled environment free of the clinical confounders to address reliably reproduced VMB that significantly altered HIV-1 infection and/or replication. This represents the first system to study lab-cultivated VMB thereby providing the opportunity to establish impact on STI outcomes and to better predict safety and efficacy of vaginal applicants.

## Results

### Vaginal epithelial cell (VEC) multilayer co-culture susceptible to HIV-1 infection

We previously reported the establishment of a stratified squamous VEC multilayer culture system with an apical air-interface that produces the necessary carbon sources for colonization by several single species commensal bacteria common to the vagina [36]. To study the impact of complex bacterial community types upon HIV-1 infection we modified the cultures to include cell types susceptible to HIV-1 infection. Two types of HIV-1-susceptible cells were selected for evaluation and optimization. TZM-bl cells, derived originally from HeLa cells by transgenic addition of human CD4 (the primary HIV-1 receptor) and CCR5 (HIV-1 coreceptor) and an HIV-1 Tat responsive b-galactosidase (b-gal) reporter system [37]–[39], were selected based on the cervical tissue origin of HeLa cells and their promiscuous growth conditions. Primary human monocyte-derived macrophages (MDM) were selected as the second susceptible cell type to allow study of a natural HIV-1 target cell that was fully competent for immune responses. Macrophages are also common to vaginal tissues suggesting the potential for co-culture with the VEC [40]. Media and culture conditions were optimized for the co-cultures in both 24 and 96 transwell formats. The 96 well format provided the opportunity for medium throughput analyses essential to future drug screening or advanced study designs.

**Figure 1:**
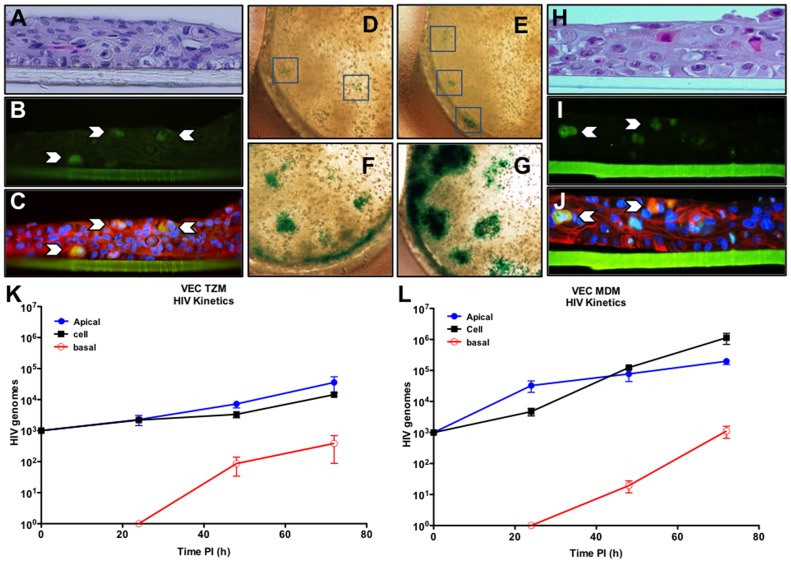
Refined vaginal epithelial multilayer co-cultures that support HIV-1 infection. Optimized conditions for establishment of co-cultures that contain HIV-1 susceptible cells are illustrated in panels A–J. Panels A–G represent outcomes with VEC/TZM-bl co-cultures as indicated by H&E staining of 6 um sections (A) or immunolabeling of a section showing the presence of CD4+ TZM-bl cells (B, arrows indicate positive cells) in the context of VEC labeled with cytokeratin and DAPI (C). TZM-bl cells provided a b-gal reporter system to detect HIV-1 infection via staining with X-gal (blue precipitate) as shown in panels D–G. Panel D shows X-gal localization of HIV-1 infection (blue box) of an uncolonized culture while E–G illustrate cultures apically colonized with clinical VBM (CSTI, CSTIII and CSTIVB, respectively). Co-cultures with primary human MDM were established as illustrated by H&E staining (H), CD4 immunolabeling ((I); green cells indicated by arrows are MDM) in the context of cytokeratin and DAPI labeled VEC (J). HIV-1_SX_ infection was evaluated over time in triplicate multilayer VEC/TZM-bl (K) or VEC/MDM co-cultures (L). The transwell culture system provided sampling of each chamber (apical in blue, cell fraction in black and basal chamber in red) representing different aspects of the viral infection process.

The presence of HIV-1 susceptible cells did not alter multilayer formation based upon morphology and thickness shown by hematoxylin and eosin (H&E) staining (Fig. 1A, TZM-bl & H, MDM). Labeling of co-culture multilayer sections with anti-CD4 antibodies showed the periodic distribution of the co-cultured CD4+ TZM-bl cells (Fig. 1B) in the VEC multilayer (Fig. 1B & C). VEC-TZM-bl wells infected with HIV-1_SX-1_ (an R5 strain that infects through CD4 and the CCR5 coreceptor expressed on both TZM-bl and MDM) were stained with X-gal (48 h pi) to localize b-gal+ cells. *In situ* X-gal staining of infected cultures provided the opportunity to identify the number and size of HIV-1 replication foci (Fig. 1D–G). Uninfected cultures showed no evidence of any blue deposition.

MDM offered many advantages for study of natural HIV-1 infection including important signaling pathways active in these professional immune cells that are inactive in TZM-bl cells. For this reason, VEC-MDM co-cultures were also optimized and evaluated as shown for VEC TZM-bl (Fig. 1H–J). For both co-culture systems, the ratio of HIV-1 susceptible cells to VEC plated in the transwells (1∶1000) was based on published estimates of normal and inflamed vaginal tissues [40] and ultimately was selected based on optimal infection outcomes for subsequent evaluations of HIV-1 replication. The data indicated that under the optimized culture conditions both TZM-bl and MDM established “tissue” niches that led to at least 14 d of viability. TZM-bl cells expanded into clusters but the MDM remained as single cells in the multilayers.

Basic viral replication kinetic profiles were established by delivery of the inoculum to the apical surface of mature multilayer co-cultures (d7 post plating) simulating vaginal exposure to HIV-1. The transwell system also provided for the opportunity to model three compartments associated with HIV-1 infection. Specifically, the co-culture apical surface was subjected to gentle lavage to collect virus associated with female to male transmission, the cell fraction was harvested to quantify active viral replication and integration and, finally, the basal compartment was sampled to measure virus that transcytosed the multilayer modeling potential spread to systemic sites. Samples were analyzed by HIV-specific quantitative RT-PCR from each compartment harvested at 24, 48, or 72 h pi. The initial inoculum of 1,000 TCID50 of HIV-1_SX-1_ (MOI of 1) led to increased genomic titers with lower amounts of virus produced in both apical and cellular fractions from VEC-TZM-bl co-cultures than was detected in VEC-MDM transwells infected in parallel (Fig. 1K & L, respectively). No virus was detected in the basal chamber of either co-culture until 48 h pi confirming the multilayer integrity. These results led us to select 48 h of infection for the future studies of the cellular fraction and confirmed the utility of both co-culture systems for study of HIV-1 infection.

### VEC co-cultures supported clinical vaginal microbiome (VMB) colonization

We previously reported that the air-interfaced, apical surface of VEC multilayers could be colonized with lab-cultivated, single species bacteria and that colonization altered cytokine secretion induced by challenge with selected TLR agonists [36]. To determine if matured VEC-TZM-bl and VEC-MDM co-culture multilayers could support colonization by intact VMB we collected mid vaginal swab samples from asymptomatic patients undergoing routine gynecological examinations. Swabs were collected into sterile PBS after consent of the participant and then sterilely processed and cryopreserved in small aliquots. Each sample was evaluated for Nugent score (NS), Amsel's criteria and then categorized molecularly into a CST [34]. Molecular evaluations were completed using a novel PCR array that provided quantitative estimations of 40 of the most relevant bacterial species or genetic elements necessary for CST profiling and BV state evaluation (Table 1). Samples that were found to contain an STI including, *Chlamydia trachomatis*, cytomegalovirus, herpes simplex virus, human papilloma virus, *Mycoplasma genitalium*, *Neisseria gonorrhea*, *Trichomonas vaginalis*, *Ureaplasma spp* or detectable yeast (*Candida spp*) were excluded from additional study. Finally, as indicated by PCR detection of the presence of Y chromosome sequences, samples from women who had recent sexual intercourse also were excluded. Using these criteria, seven VMB representing 5 of the 6 CST and a range of NSs were selected for testing (Table 1).

**Table 1:** Quantified genomes of selected bacteria in representative VMB communities in colonized VEC multilayer cultures relative to the inocula.

TARGET	CST I (NS0)		CST II (NS1)		CST III (NS2)		CST III (NS5)		CST IVA (NS5)		CST IVB (NS8)		CST IVB (NS8)	
	Inoc	48 h	Inoc	48 h	Inoc	48 h	Inoc	48 h	Inoc	48 h	Inoc	48 h	Inoc	48 h
**Human GAPDH**	4	6.3±5.6	2.38	6.8±6.6		6.9±1.5	4.21	6.5±5.9		5.8±5.1		7.1±7	2.99	6.5±6.3
**Total bacteria (16S)**	6.06	6.1±5.9	6.26	7.03±6.1	7.86	8.9±4.9	5.82	7.3±7.3	7.8	8.04±3.1	7.86	8.99±4.6	7.83	8.4±7.4
***Lactobacillus spp***	4.22	5.35±5.3	2.23	5.45±4.3	6.65	7.85±7.4	4.06	7.17±5.6	5.5	5.75±5.6	2.79	4.94±3.9	3.81	6.63±5.6
***L. crispatus***	5.04	5.06±4		3.13±3			6.68	4.84±4	4.8	5.59±4.8				5.27±4.5
***L. gasseri***			3.85	5.86±4.9										
***L. iners***	3.66	4.17±3.1			5.29	4.66*		6.96±6.2	5.04	5.3±5.1				
***L. jensenii***	2.78	4.41±3.6				2.39*		5.7±4.8	3.74	3.48±2.8				3±2.94
***L. fermentum***			2.59	3.77±3.6										
**Acidovorax**	2.21	3.77±3.5	2.53	3.5±2.8	3	3.64±2.1	3.7	3.74±2.8	3.08	3.24±2.4	2.7	3.76±3.9		3.94±3.9
**Aerococcus**								3.56±2.7				4.32±3.25		6.73±5.7
***Atopobium vaginae***		4.87*				4.66±3.8		4.95±3.5				6.49±6	4.13	5.70±4.7
**BVAB1**													4.1	5.57±4.5
**BVAB2**													4.1	4.93±4.6
**BVAB3**													4.0	4.2±3.9
**BVAB-TM7**													4.1	4.95±4.8
**Cloacibacterium**				2.09±1.9	2.74		3.7	2.8±3	2.51	1.63±1.5	2.7	3.4*	2.34	2.7±1.6
**Corynebacterium**	3.71	4.04±3.9	1.4	3.01±2.1		4.22±2.6	4.11	5.24±4.2	4.45	4.52±3.4	2.94	2.64±2.8	2.96	6.42±5.6
**Dialister**						3.73*				3.77±3.6		4.23±3.9		4.18±3.9
***E. coli***				4.54±2.4						3.06±3				
**Eggerthella-like**														3.78±3.3
**Enterococcus**	3.84	3.37±2.2	2.27	2.56±1.7		5.92		5±4.2	3.43	3.43±3.2	3.24	4±3.9	4.1	7.43±6.5
***Gardnerella vaginalis***						6.11*						7.12±6.6	4.1	7.1±6.2
***G. vaginalis*** ** sialidase**						5.7±4						5.94±5.9	4.1	6.12±6
**Haemophilus**		4.14±3.8	1.64	1.24±0.4		3.75±3.1		4.08±3.6		2.77±1.6		3.74±3.5		3.89±3.3
**Lachnospiraceae**									4.81	3.22±3.1		3.9		
***Leptotrichia amnionii***		3.64*	1.16					3.93±4		2.06±1.9			4.4	5.68±4.6
**Megasphaera Type 1**												5.42±4.4		4.43±3.7
***Mobiluncus mulieris***													4.1	4.76±3.7
***Mycoplasma hominis***						2.11*								1.37*
**Parvimonas**														3.44±3.1
***Peptoniphilus lacrimalis***						2.67*								4.7±3.7
**Peptoniphilus**						2.81*								4.5±4.3
**Peptostreptococcus**														3.89±3.6
***Prevotella buccalis*** **-like**													4.1	3.5±3.3
**Prevotella G1**														2.88±2.6
**Prevotella**						3.86*				2.47±2.3		4.33±4.2	4.10	6.27±5.6
**Proteobacteria a, b**	2.01	3.14*					4.26	4.1±3.3	3.53	3.3±3.1		4.71±4.5	3.54	3.75±3.7
**Proteobacteria b, g**	2.26	4.6±4.4	3.1	3.6±3.7	4.32	4.24±0.1	5.06	4.6±3.5	4.15	3.5±3.3	4.06	4.6±4.5	3.93	4.6±4.5
**Ruminococcaceae**		1.3*			3.18	4.1±0.1	4.02	3.56±2.8						3.95±4.1
***Sneathia sanguinegens***		4.11*						4.08*				3.32*	3.88	6±5.3
**Staphylococcus**						3.31*		4.32±3.9					3.8	6.22±6
**Group B Streptococcus**				4.61*				5.47±4.5		6.44±6.2	5.05	5.62±5.5		1.84±1.6
**Streptococcus**		4.06±3.9	2.62	3.21±3.3	3.81	7.41±6.6		3.8±3.7	3.49	6±5.6	3.33	5.86±5.6	3.4	4.7±4.6
**Veillonella**						3.63*		3.53*		3.04±2.8				3.22±2.3

The log_10_ average genomic count (N = 6–9) established by qPCR for the indicated bacterial genus or species ± the standard error of the mean are depicted. Empty cells indicate a lack of detectable target DNA in each sample. Values marked by “*” indicate only a single positive value was detected among the sampled cultures in that group. A portion of each undiluted inocula (inoc) of roughly equivalent volume was quantified in parallel to the bacterial levels present 48 h after colonization of the matured VEC multilayers.

Aliquots of the selected samples were thawed and tested for general bacterial viability by creating a 10-fold dilution series in Mann-Ragosa Sharpe (MRS) broth to quantify bacterial titer [36]. Using optimized cryopreservation methods, viable bacteria were observed at titers of ∼10^5^–10^8^/ml in the thawed samples and were similar to the titers established in the freshly collected, original swab sample. We next evaluated whether these cryopreserved VMB samples could be used to colonize the apical surface of established VEC multilayer cultures. Thawed samples were diluted 1,000- to 100,000-fold in PBS and apically applied to mature co-cultures and molecularly followed for increases in bacterial titers and preservation of community diversity (Table 1). The target inoculum, based on total bacterial genomic load measured by 16s rDNA qPCR, was 1,000–5,000 bacterial genomes. The data, summarized from three distinct studies, showed reproducible community establishment for each of the selected CST independent of the type of co-cultured multilayer (Table 1). Uncolonized cultures were prepared in parallel for every study and consistently tested negative for any bacteria. It is notable that several previously uncultivated bacterial species (e.g. BVAB species, CST IVB NS8) were propagated in this format. Additionally, in the context of complex VMB populations, *L. iners* colonized the VEC co-cultures (e.g. CSTs I, III and IVA) in contrast to our previous inability to viably colonize multilayers with this single species [36]. Consistent with these collaborative interactions, the colonized multilayer cultures supported many species of anaerobic bacteria in the 5% CO_2_ supplemented normal air environment consistent with the natural environment created in the human vagina.

**Figure 2:**
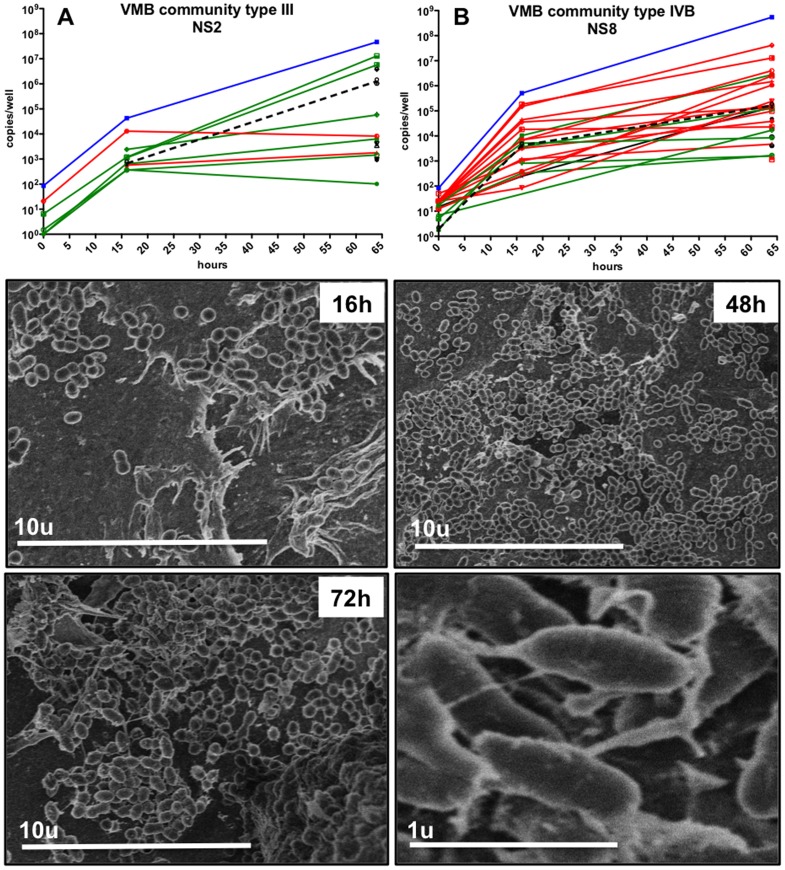
VMB colonization of VEC/TZM-bl multilayer co-cultures recreated the complex bacterial communities present in clinical samples. To confirm that the environment created by VEC multilayer cultures could support colonization by in tact VMB, established cultures in 96 well format (N≥6/time point) were inoculated apically with ∼1,000 bacterial genomes and followed kinetically by qPCR (A and B). A representative VMB of CST III (Nugent score, NS 2) and a CST IVB (NS8) types are depicted to illustrate that overall bacterial load (blue line) increased over the 64 h evaluation with a coincident increase in host genomes (black line) indicating culture health. In both graphs, green lines represent bacteria associated with healthy vaginal status and red lines are considered pathogenic. Quantified data for the bacteria targeted by the qPCR from these and other representative VMB are presented in Table 1. The lower panels depict SEM evaluations of a set of parallel cultures (2/time point) colonized by a CST IVB VMB with community diversity. Colonized cultures were fixed at the indicated times and visualized to illustrate the increasing number and diversity of bacterial morphotypes as well as the production of extracellular polymeric substances coating and connecting individual bacteria (lower right panel).

Two VMB communities of intermediate (CST III, NS2) and extreme population diversity (CST IVB, NS8) were selected to illustrate the quantitative increase in multiple species as shown Table 1 and as depicted graphically in Fig. 2A & B, respectively. In parallel with the molecular evaluations, colonization by the highly complex CST IVB VMB was followed kinetically by scanning electron microscopy (SEM) to visualize the increases in both total bacterial number and morphotypes (Fig. 2, 16–72 h). At higher magnification the presence of extracellular polymeric substances coating dense areas of mixed morphotypes of bacteria indicative of biofilm formation at 48 and 72 h after colonization (Fig. 2C, bottom right). Collectively these data provided two essential outcomes. First, the kinetic assessments confirmed that an optimized cryopreservation method was established that preserved viable diversity of the VMB communities allowing for repeated study of the same VMB. As shown in Table 1, all of the outgrowth profiles fell into the same CST as the original inoculum however, there were some noted diversity changes as described below. The current data suggest that stable cryopreservation of at least 5 years is possible. The second important outcome was apical application of VMB samples confirmed that the VEC co-culture multilayers were capable of supporting colonization by these complex bacterial communities in reproducible fashion (n≥3 studies). Importantly, these cultures provided the first lab-based cultivation of many of the bacterial species previously only studied by cultivation-independent methods including the bacterial vaginosis-associated bacteria (BVAB) species and several other anaerobic species.

To identify limitations of the VEC culture system, we evaluated colonization by multiple VMB looking for specific genus or species-level community members that consistently failed to increase in titer. The vast majority of identified community members increased or maintained genomic titers relative to the levels predicted by the original inoculum (Table 1). In fact, several bacterial species undetected in the inoculum were detected after 48 h of colonization (e.g. *Peptinophilus spp*, group B streptococcus, *Aerococcus spp*, *Dialister spp*, and *A. vaginae*) suggesting that for such bacteria, the VEC multilayer provided a potentially enhanced environment relative to the vaginal mucosa. Similarly, a few bacteria maintained or had slightly lower titers or were lost from the population at 48 h in a community dependent fashion (Table 1; e.g. *Prevotella buccalis*-like, *Cloacibacterium spp* and *Proteobacteria spp*). Unexpectedly, in the tested CST III VMB, *L. crispatus* failed to thrive in contrast to our data from single species colonization [36].

We also assessed the inflammatory state established by each of the CST representatives and as previously observed with commensal single species [36], no detectable increase in secreted cytokines in apical washes or in cell fractions from VEC co-cultures colonized was produced by any CST other than the III or IVB representatives (Table 2). Colonization with a CST III or IVB representative led to significant increases in IL-1b, IL-8, TNFa, RANTES and IL-1ra (Table 2). There was a notable but not significant trend for the CST I and II representatives to reduce baseline secretion of selected cytokines consistent with our previously reported findings with single *Lactobacillus spp* colonization [36].

**Table 2:** Cytokine secretion from VECMDM multilayers colonized with selected bacteria or VMB CST.

Cytokine	No bacteria	*L. crispatus*	CST I (NS0)	CST II (NS2)	CST III (NS5)	CST IVB (NS8)
**IL-1b**	9.2±0.4^a^	2.3±0.6	0.9±0.4	4.8±0.8	40.6±13.9*	112.4±18.3*
**IL-6**	0.2±0.02	1.3±0.5	0.3±0.1	0.3±0.1	11.9±2.4	3.5±1.2
**IL-8**	4.1±0.8	5.9±1.6	6.8±2.4	7.7±3.9	194±34*	4560±808*
**TNFa**	5.3±0.9	7.8±2.5	10.5±2.7	5.1±1.1	310±119*	158.7±48.9*
**G-CSF**	24.5±2.5	27.6±3.4	28.1±8.2	25.7±5.2	35.9±3.2	86.3±5.2
**GM-CSF**	6.6±2.3	2.9±0.5	2.3±0.6	12.7±5.8	2.9±1	47.6±11.7
**RANTES**	10±0.8	4.4±0.8	2.5±0.8	1.7±0.5	45.1±11.1*	52.3±5.1*
**IL-1ra**	768±319	494.7±42.3	576±149	590±69	4594±761*	6493±1671*

Single species bacteria or representative VMB's were inoculated on the apical surface of mature VECMDM multilayers at ∼1000 genomes. Cell fraction sampling was completed at 48 h after colonization.

a Selected cytokines are presented as mean+SEM (pg/ml) of 3 replicates from 2 independent studies.

*p<0.05 compared to no bacteria (Student's t-test).

### VMB altered HIV-1 titers showing both suppressive and enhancing communities could be identified

**Figure 3:**
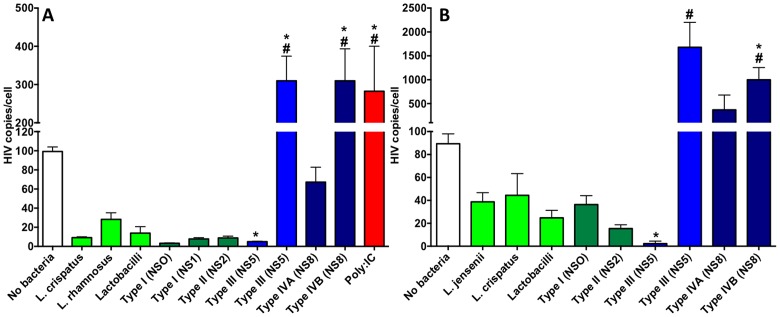
HIV-1 burden was significantly altered by VMB colonization of VEC multilayer co-cultures. To directly study the impact of representative VMB upon HIV-1 infection and replication VEC/TZM-bl (A) or VEC/MDM (B) co-culture multilayers were established in 96 well format. Matured cultures were colonized and virally challenged as described in the methods. Representative healthy (dark green bars) or pathogenic (blue bars) VMB, single species bacteria or the probiotic Lactobacilli mixture (light green bars) are indicated on the x-axes. Mean genomic HIV-1 titer is shown on the y axes. Statistical comparisons were completed by one-way ANOVA with Bonferroni's or Dunnett's multiple comparison correction. A “*” indicates a significant difference (P<0.05) relative to the uncolonized viral burden. A “#” indicates a significant difference (P<0.05) with the suppressive CST III VMB. Triplicate cultures were created and the study was replicated twice. Poly-I:C (0.1 mg/ml) was utilized as an inducer of HIV-1 infection and replication and was applied at the time of colonization.

Based on the successful colonization of VEC co-cultures and successful HIV-1 infections of the co-cultures, we tested the hypothesis that colonization by the VMB CST representatives would alter HIV-1 infection/replication as indicated by genomic titers in the cellular fractions. Three replicate studies were completed and the outcomes are presented as average HIV-1 genomic burden in the context of uncolonized or specifically colonized VEC co-cultures established with either TZM-bl or MDM (Fig. 3A and B, respectively). For the first studies in VEC-TZM-bl cultures, poly-inosine∶cytidylic acid (poly-IC) was applied to uncolonized multilayers 16 h before HIV-1 infection to enhance HIV-1 replication relative to untreated, uncolonized control cultures (Fig. 3A red bar vs. white bar, respectively). Poly-IC significantly increased HIV-1 genomic titers in treated cultures (p<0.05) and provided an indication of potential enhancement of viral replication in the co-culture system. As indicated by the blue bars (Fig. 3A), genomic titers, similar to those in Poly-IC-treated co-cultures, were produced in VMB with higher NS (CST III, IVA and IVB). Consistent with the data shown in Fig. 1, VEC-TZM-bl co-cultures produced less HIV-1 than VEC-MDM co-cultures (Fig. 3A vs. B) but following VMB colonization extremely similar patterns of viral burden were observed in a community-dependent fashion. Both types of co-culture indicated that, relative to uncolonized cultures, one of the two selected CST III communities (containing at least 17 distinct community members dominated by *L. iners* and group B streptococcus; Table 1) significantly suppressed HIV-1 replication (Fig. 3, p<0.05). Similarly, although not significantly different from uncolonized cultures, HIV-1 levels in all of the CST I, CST II VMB or single species Lactobacilli-colonized VEC-TZM-bl and VEC-MDM co-cultures trended lower indicating HIV-1 suppression associated with these bacteria. Additional confirmation was provided by X-gal staining of infected, colonized VEC-TZM-bl co-cultures that showed small foci (Fig. 1E) relative to VMB that produced higher HIV-1 burdens (Fig. 1F and G).

Interestingly, the second CST III community (NS5), collected ∼60 d earlier from the same woman as the most suppressive VMB, showed a distinct community profile that was dominated again by *L. iners* and group B streptococcus but included at least 4 additional bacterial species that contributed to enhanced HIV-1 replication (p<0.05, Fig. 3). These bacteria included *Ruminococcaceae spp*, *Aerococcus spp*, *Sneathia sanguinegens* and *Atopobium vaginae* (Table 1). These organisms were present at similar or higher levels in the CST IVB community that also produced significantly higher HIV-1 levels relative to uncolonized controls (p<0.05). Comparisons of the two VMB that significantly enhanced HIV-1 genomic titers to those produced in each of the CST I, CST II VMB or single species colonized co-cultures (green bars) also were significantly different (p<0.05). Of the 4 bacteria common to the HIV-1 enhancing communities, CST I and CST II communities had lower but detectable levels of *Ruminococcaceae spp*, *S. sanguinegens* and *Atopobium vaginae* (Table 1). *Aerococcus spp* was the only identified bacteria absent from the suppressing VMB types (Table 1). Study of additional community representatives for each CST will be required to complete comprehensive associations but at present these data indicate the utility of the model system and establish the potential for subsequent evaluations of additional clinical samples.

### Multilayer colonization by VMB impacted tenofovir (TFV) efficacy and illustrated unexpected alterations in the VMB composition

**Figure 4:**
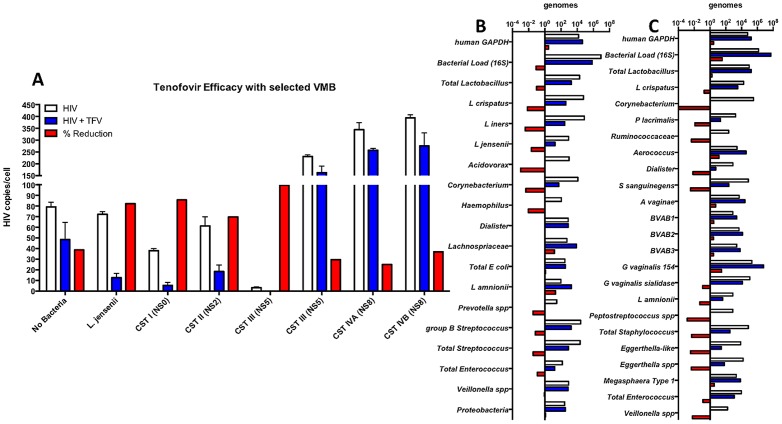
TFV efficacy was altered by representative VMB colonization and impacted the composition of VMB. Panel A depicts the anti-HIV-1 efficacy of TFV in VEC/TZM-bl cultures (N = 3) colonized as indicated with representative VMB. The white bars indicate HIV-1 titers in untreated parallel cultures and confirm the previous results (Fig. 3). The presence of TFV (blue bars) consistently reduced the numbers of viral genomes but, as shown in the red bars, a non-significant trend indicated that the level of reduction was VMB-dependent. Panels B and C, respectively, depict the most suppressive and most enhancing VMB community profiles in the absence (white bars) or presence (blue bars) of TFV (N = 2 cultures/condition). The fold change in detected bacterial genomes associated with TFV application is shown for each of the selected organisms as a loss (red bar on left of the vertical axis) or gain (right of axis) in number relative to the untreated cultures.

Clinically, VMB appear to alter the efficacy of vaginally-applied antiretroviral compounds through unknown mechanisms. Conversely, molecular evaluations have indicated that selected vaginal applicants alter the composition of VMB [20]. Therefore, we sought to evaluate the impact of a single dose of the FDA-approved antiretroviral TFV delivered to colonized cultures just prior to HIV-1 challenge. The results showed that TFV-treated cultures had reduced HIV-1 burdens relative to untreated cultures (Fig. 4A, blue versus white bars). As a measure of efficacy, we calculated the percentage of HIV-1 genome reduction by dividing the difference in HIV-1 load in TFV-treated and untreated cultures by the average HIV-1 burden in the untreated cultures (Fig. 4A, red bars). Although not significantly different, there was a trend indicating reduced TFV efficacy in the cultures colonized by the HIV-1 enhancing VMB relative to those with suppressing communities. Specifically, the CST III VMB that was most suppressive had a 99% reduction in HIV-1 burden following TFV treatment. The CST IVB community that increased HIV-1 titers most significantly, had only an average 37% reduction in HIV-1 after TFV. This level of efficacy was similar to uncolonized TFV-treated cultures. The lowest TFV efficacy, only 25% reduction in HIV-1, was observed in the CST IVA community that had similar HIV-1 enhancement to the IVB community.

A limited preliminary study of the impact of TFV upon VMB composition was completed to determine if the colonized co-culture system could be used to complete in vitro assessments of potential VMB toxicity. For this evaluation the profiles of the suppressing CST III VMB from a pool of three cultures treated with TFV or three parallel cultures that were treated with vehicle alone were compared (Fig. 4B). Similarly, the HIV-1 enhancing CST IVB community also was assessed (Fig. 4C). Consistent with studies by members of our team that indicated biofilm formation and thickness on the surface of emplaced intravaginal rings (IVR) were not generally altered by TFV [41], the overall bacterial load in colonized co-cultures for both CST VMBs was not significantly different in the presence or absence of TFV. Further, as generally observed in the CAPRISA 004 data sets and early topical TFV safety testing [42], [43], the levels of total Lactobacilli were maintained or reduced by ∼10 fold by TFV application. Interestingly, individual Lactobacilli species were substantially reduced in number in the suppressive CST III VMB (Fig. 4B, red bars, ∼100 fold) suggesting Lactobacilli species not included in our qPCR assays were increased. Reductions in levels of other bacteria in the CST III VMB included Acidovorax, Corynebacterium, Haemophilus, *Prevotella spp*, total Streptococcus and total Enterococcus. A complete loss of Corynebacterium was observed in the TFV-treated CST IVB cultures. Reductions in total Enterococcus also were observed as were a number of additional bacteria common to the CST IVB community including Dialister, *S. sanguinegens*, *Peptostreptococcus spp*, total Staphylococcus, *Eggerthella spp* and *Veillonella spp*. TFV treatment increased a few bacteria types within each community but generally to a lesser degree than seen with the reductions. In the CST IVB community, a single application of TFV treatment was associated with increased levels of *Aerococcus spp*, *A. vaginae*, the BVABs, and *G. vaginalis*. Paradoxically, the amount of *G. vaginalis* sialidase DNA was reduced. Collectively, these preliminary studies suggest the VMB colonized multilayer co-culture model will be of substantial use for prediction of vaginal applicant impact upon the VMB but additional association with clinical data will be required.

## Discussion

There has been a pressing need to develop a model system to study the intact VMB in the context of VEC and professional immune cells. Since the earliest descriptions of the bacteria that colonized the human vagina there have been many efforts to cultivate the complex communities in the laboratory. Recent success with cultivation-independent approaches has established an accurate picture of the community members and the impact of external influences upon the VMB [20], [31], [33], [34], [44] but each of these studies has been complicated by the genetic diversity of the human participants and the nature of the sampling. These factors have substantially impacted the development of statistical power thereby limiting the quality of the conclusions. We present the results from a combination of recent advances in cell culture systems that create more accurate models of the vaginal mucosa [36] with methods to molecularly evaluate and reproducibly propagate intact vaginal communities recovered from vaginal samples collected during routine gynecological exams. The system provides the first lab-based opportunity to begin the study of the important species or partnerships between species in both healthy and dysbiotic VMB.

Our VEC multilayer culture system provides the necessary environment to support colonization by intact, transplanted VMB representing 5 of the 6 established CST (Table 1). The current data indicate that VMB established in our cultures retained the general character of the VMB detected in the clinical inocula but there were noted differences in some bacterial levels. In some cases, these differences are most explained by a lower than detectable level of specific bacteria in the inoculum that expanded in the favorable environment created by the VEC multilayer. Conversely, as shown by the representative CST III (NS5) and IVB VMBs several community shifts (e.g. *L. iners*) could be seen consistently in the established VMB. Further studies will be required to evaluate the impact of such differences that have been widely reported in clinical studies of the same woman over time (20, 33, 34). The reproducibility of the established VMB indicates the utility of the culture system but may reveal a limitation of the cultures with regard to certain community profiles. Further CST representatives will need to be tested to more fully validate the VEC multilayer system.

Cryopreservation of the VMB sample allowed for repeated analyses and development of mechanistic studies to understand the relationships formed between bacterial community members and between the VMB and the underlying host cells. This was completed in a defined genetic background provided by the immortalized VEC and controlled culture conditions. Further, the model provides opportunity for controlled introduction of influences that would not be possible in clinical research settings. The refinement provided by the established co-cultures also enables the opportunity to begin to study the molecular dialogs between professional immune cells common to the vaginal mucosa and the surrounding VEC in the context of controlled colonization by selected VMB. Such studies will include evaluation of the metabolome and signaling molecules common to each VMB. Two elegant clinical evaluations were recently reported showing the metabolome and proteome of vaginal fluids collected from women before and after treatment for BV that identified a number of metabolic markers and signaling molecules associated with BV [45], [46]. Controlled addition of specific immune cells in the context of selected colonization should help advance these types of studies in the future.

The established co-cultures also allow for study of STI outcomes in the context of the VMB including life-threatening HIV-1 transmission and infection as described in this report. It has been estimated that HIV-1 infection requires 200–2,000 exposures to virus during vaginal intercourse but during BV episodes and coincident inflammation in the vaginal mucosa the risk of HIV-1 transmission is significantly increased [21], [47]. Clinical associations have established that these time periods include significantly elevated levels of pro-inflammatory cytokines including TNFa, IL-1b, IL-6 and IL-8 [48], [49]. Consistent with these clinical findings, the VEC co-cultures colonized with intermediate or high NS VMB of CST III or IVB led to significantly elevated production of IL-1b, IL-8, TNFa, as well as RANTES and IL-1ra (Table 2). IL-6 trended higher in both community types but was not significantly different than uncolonized cultures. These data are in line with a clinical study that concluded IL-8 but not IL-1b or IL-6 were associated with BV [50]. Coincident with these elevated levels, we found that these communities led to higher HIV-1 burden and reduced efficacy of a single application of TFV (Fig. 3 & 4). Similar cytokines are elevated by several failed vaginally-applied candidate microbicides including nonoxynol-9 and cellulose sulfate that led to increased susceptibility to HIV-1 infection [20], [28]. In the culture system, elevation of these cytokines likely led to an activated state in the MDM more favorable for HIV-1 replication but additional research will be required to establish the mechanism for the enhanced or suppressed HIV-1 outcomes.

The application of next generation sequencing has also recently shown that the vaginal application of candidate microbicides and other products altered the composition of the VMB of treated women. These studies mark the beginning of improved toxicity screening of candidate compounds but are expensive, labor-intensive and rely upon clinical materials emphasizing the need for a model system capable of screening vaginally-applied compounds for impact on the VMB prior to clinical evaluation. As proof of concept, we tested the impact of a single application of TFV upon HIV-1 in the context of colonized cultures and also assessed the community changes (Fig. 4). Although limited in scope, the data indicated that the efficacy of TFV was reduced by some of the selected VMB community members. Another limitation of our approach was the use of qPCR assessment of the VMB composition limiting the list of bacteria studied to those targeted by the assays. Clearly there are bacteria that are not accounted for that would be identified by the more costly and labor intensive next generation sequencing approaches. Additional studies will be required to complete the analyses of TFV but the current data indicate the potential utility of the system.

Recent clinical trials of topical vaginal microbicides designed to reduce HIV-1 transmission including CAPRISA 004 have shown the potential of a TFV gel formulation. The impact of vaginally-applied 1% TFV gel on VMB community composition has not been reported but in clinical trials that included assessment of the microflora there were indications of altered community profiles based on Nugent scoring [43]. No significant differences in BV-related symptoms (e.g. vaginal discharge) were reported between TFV-treated and placebo groups [42], [43]. Consistent with these outcomes, a single application of TFV in our culture system led to disruption of the community profile for both the healthy and BV VMB selected for evaluation (Fig. 4). In both of the analyzed VMB the overall bacterial load was generally preserved but loss of distinct species and overgrowth by others was noted (Fig. 4). Deep sequencing data sets from clinical samples representing TFV treated versus placebo controls are needed to validate the predictions of the culture data but are consistent with the changes in reported NSs [43]. The data from the culture system lack the confounders associated with clinical sampling and strongly support additional evaluations of vaginal applicants for impact upon the VMB prior to clinical trials.

The culture system also provides the first opportunity to study bacterial interactions, products and signaling during the formation of the pathogenic biofilms associated with BV. Other cell culture models have been used to evaluate the initial adhesion and potential order of introduction of bacterial species that overwhelm otherwise healthy VMBs [51], [52]. Reports from the human vaginal microbiome center and collaborating researchers have shown how BV-associated bacterial species that can be cultivated in the lab adhere to HeLa or ME-180 cell line derived from cervical cancers [51], [52]. These recent reports suggest that *G. vaginalis* is an initial colonizer setting up for more damaging VMB and biofilm formation [51], [52]. Although these study designs lack the intact VMB and impact of existing partnerships on colonization order, they provide advantages of data created from evaluation of individual species. A limitation of our current study is the composition of the clinical VMB that we have selected and the limited numbers of species represented. We previous reported colonization of VEC multilayer cultures with selected single Lactobacillus species or other bacteria common to the vagina showing that commensal bacteria associated with health tempered innate immune responses [36].

The VEC multilayer model also provided three compartments that each represent specific aspects of vaginal tissue as well as support of study of the HIV-1 infection cycle. Using HPV E6/E7 oncoprotein immortalized rather than cancerous cells has provided the opportunity for expanded numbers of available cells of the same genetic background without the issues associated with oncogenic transformation. The immortalization process does alter the cell's physiology but the expansion limitations and difficulties associated with tissue procurement to create primary cells require the use of such cell cultures for initial screens. Subsequent confirmation studies with cryopreserved primary progenitors are warranted for some candidate evaluations.

During our foundational evaluations of the culture system we intentionally avoided the addition of exogenous hormones essentially modeling the transition between the luteal and follicular phase with the VEC co-cultures. Future work will include the addition of progesterone and/or estrogen to reflect levels associated with specific stages of the menstrual cycle. Subsequent studies will allow for the evaluation of components of seminal fluid, over the counter vaginal applicants and ultimately provide a system to evaluate the potential toxicity of candidate compounds and probiotics.

## Materials and Methods

### Overall study design

The goal of the designed studies was two-fold. The first experiments were completed to establish the potential of the VEC culture system and to create data sets that showed the reproducibility and consistency of VMB colonization from cryopreserved clinical material. The second phase of the study design was undertaken to establish the impact of selected CST representative VMBs upon HIV-1 replication in the refined VEC co-culture models. The resulting culture system also provided preliminary data for the impact of a single dose of TFV on HIV-1 replication and on the representative VMB community composition. Collectively, the data show that the culture system provided an effective environment to support colonization by the diverse and highly complex bacterial communities associated with both health and dysbiotic BV states. Controlled replicate cultures were established to study the VMB effect upon HIV-1 infection independent of host genetics, hormonal or environmental factors, yeast, the presence of other STI or genital pathogen or by the natural fluctuations of the VMB observed in recent cultivation-independent evaluations. The presented data represent results from studies in VEC from a single genetic background under highly controlled conditions but have been completed in limited fashion in two additional cultures with similar outcomes.

### Cell culture

Immortalized V19I VEC were cultured as described previously [36], [53]. TZM-bl cells are a HeLa-derived, genetically engineered HIV-1-susceptible cell line that provided the advantage of a b-galactosidase (b-gal) reporter system induced by HIV-1 Tat. TZM-bl cells were obtained through the AIDS Research and Reference Reagent Program, Division of AIDS, NIAID, and NIH, from Drs. Kappes and Wu and Tranzyme Inc. [39] and were cultured as described [37], [38]. Monocyte-derived macrophages (MDM) were established from peripheral blood collected from healthy volunteers with full IRB approval as previously described [53]. For these studies data from a single donor are presented but were consistent across four distinct donors. Fully mature MDM were differentiated within 7 d of conditioning and were harvested in parallel to VEC for establishment of co-cultures. Co-cultures were created by mixing VEC with the selected HIV-1-susceptible cell type at a ratio of 1,000 VEC to 1 HIV susceptible cell just prior to plating in transwells. For the 96 well transwell format (BD Falcon; Franklin Lakes, NJ), 10^5^ cells were plated while 10^6^ cells were plated in 24 well format transwells (Greiner Bio One; Monroe, NC). Each culture was allowed to stabilize at 37°C, 5% CO_2_ for 16 h before the apical medium was removed to create the air-interface. Basal chambers were refed with antibiotic-free medium every other day as described [36]. Cultures had fully formed multilayers within 7–9 d.

### Single species and VMB community preparation and application

Indicated bacterial strains were cultured as described [36] and added at 10^3^ in phosphate-buffered saline (PBS) after extensive washing to remove the bacterial medium. Inocula were either 50 µl for a 24 transwell or 10 µl for a 96 well transwell. Clinical samples were diluted in sterile PBS based on the total bacterial genomic titer in the clinical sample as determined by 16S rDNA PCR.

To collect VMB, healthy women undergoing routine gynecological examinations were informed of the scope of the study and with their consent were sampled by gentle rubbing of a sterile calcium alginate swab on a mid vaginal wall location. Vaginal swabs were immediately placed into sterile PBS and transported to the processing lab on ice. All work was completed with care to protect the identity of the patient with full approval of the UTMB IRB. Vaginal samples were aliquoted for standard DNA extraction and cryopreservation in a sterile glycerol solution before storage at −80C. Molecular evaluations of the VMB composition were completed by PCR (Table 1 and Table S1) targeting 40 relevant species.

Bacterial viability was quantified by addition of 10 ul of the inoculum to 90 ul Mann-Ragosa Sharpe (MRS) broth (BD Falcon) followed by serial 10-fold dilution and culture at 37°C for 48 h. Bacterial growth in each dilution was assessed and allowed for estimation of the viable titers of bacteria that were supported by this broth culture.

### Ethics Statement

Collection of VMB samples and creation of MDM populations from venous blood collected from health volunteers was complete with the full approval of the University of Texas Medical Branch's institutional review board. Only adult subjects were enrolled in the study and provided written informed consent prior to collection of materials.

### HIV-1 infection, titer determination and tenofovir (TFV) efficacy

HIV-1 (1,000 TCID50) was added to multilayer cultures 16 h after colonization by the indicated bacterial strains or VMB communities. A titered stock of HIV-1_SX-1_ a chimeric M-tropic virus (R5) encoding the majority of the HIV-1_JRFL_ envelope protein in an HIV-1_NL4-3_ backbone (purchased from the Virology Core Facility, Center for AIDS Research at Baylor College of Medicine, Houston, TX) was diluted to create an MOI of 1 relative to the susceptible cells in the co-culture [53]. TFV (a gift of Oak Crest Institute of Science) was prepared as a 1 uM stock in VEC culture medium and applied as a 10 ul apical or 25 ul basal dose designed to deliver 10× the in vitro IC50 [54]. TFV application was 10 minutes prior to challenge with HIV-1 and 24 h after VMB colonization. Infected multilayer cultures were incubated at 37°C for up to 72 h and sampled as indicated. Apical samples were harvested by gently washing the surface of each culture with 100 µl of PBS with a sterile pipet. Cell layers were scraped into 200 ul of a lysis solution compatible with protein analysis or nucleic acid extraction. Basal medium was collected by sterile pipet and mixed with protein or nucleic acid extraction solutions. RNA was purified with an Aurum 96 extraction system (Bio-Rad, Hercules, CA). DNA was extracted by magnetic particle systems from Thermo-Kingfisher (ThermoFisher Scientific, Waltham, MA) in 96 well format on a BioSprint automated extraction system (Qiagen, Valencia, CA).

### Microscopic evaluations of multilayers and VMB

For histology and immunolabeling studies, multilayers were fixed in 2% formaldehyde for 10 minutes at 4°C, then washed with PBS. Wells were then incubated with X-Gal buffer [55] containing X-Gal (1 mg/ml) for 48 h at 37°C then washed with PBS before photomicroscopy was performed. For standard histological staining, membranes, removed from the transwell insert using a scalpel, were embedded in paraffin and then sectioned (6 um) and stained with hematoxylin and eosin before being imaged [36]. Immunofluorescent labeling for specific proteins was performed using an anti-human CD4 antibody (BD). Labeled sections were cover slipped with hard set containing DAPI (Vector Labs, Burlingame, CA) before imaging on a Nikon Eclipse Ti, Nikon. Scanning electron microscopy was completed as previously described [36]. Briefly, fixed cultures were dehydrated in ethanol and processed through hexamethyldisalazane followed by air-drying before mounting onto metal stubs and sputter-coated with iridium in an Emitech K575x Sputter Coater (Emitech, Houston, TX) at 20 mA for 20 sec. The filters were examined in a Hitachi S4700 SEM (Hitachi High Technologies, Schaumburg, IL) at 2 kV.

### Cytokine quantification

Triplicate mature multilayer cultures were harvested 48 h after application of *L. crispatus* or the indicated VMBs (Table 2) and compared to uncolonized control cultures processed in parallel as previously described (36). Briefly, the cell layer was scraped into a commercial lysis solution (Bio-Rad) and processed following the instructions for the human 27-plex kit (BioPlex, Bio-Rad). Approximately 1/3 of the cell fraction was analyzed and the entire study was independently repeated providing average cytokine levels for 6 distinct cultures.

### Statistics

All experiments with the exception of those presented in Fig. 4, were completed at least three times with each condition represented as at least a triplicate within an individual study. Mean values are presented with SEM values. N indicates the number of biological replicates in each study. Most data were evaluated using ANOVA tests with Bonferroni's or Dunnett's multiple comparison corrections as indicated in the figure legends. A P-value of <0.05 was considered to be significant and is indicated by a * or # symbol in the figures. All statistical evaluations were completed with Prism software (v6.0; GraphPad, San Diego, CA).

## Supporting Information

Table S1 **PCR target data for the analysis of the VMB.** Sequences for the primers utilized for the quantification of bacteria present in the clinical and lab-based samples are shown along with associated source references as indicated in the far right column. The VMB-target, GenBank accession number and the name of the target gene are provided for each of the bacterial targets included in the array.
(DOCX)
