# Supplementary material for: Cultivated Vaginal Microbiomes Alter HIV-1 Infection and Antiretroviral Efficacy in Colonized Epithelial Multilayer Cultures
Source: PLoS One. 2014 Mar 27;9(3):e93419. doi: 10.1371/journal.pone.0093419 (PMC3968159; doi:10.1371/journal.pone.0093419)
Supplement: Table S1 — PCR target data for the analysis of the VMB. Sequences for the primers utilized for the quantification of bacteria present in the clinical and lab-based samples are shown along with associated source references as indicated in the far right column. The VMB-target, GenBank accession number and the name of the target gene are provided for each of the bacterial targets included in the array. (DOCX) [file pone.0093419.s001.docx]

| **VMB-Target** | **Accession** | **Target** | **Forward Primer (5’-3’)** | **Reverse Primer (5’-3’)** | **Reference** |
| --- | --- | --- | --- | --- | --- |
| Acidovorax | KC197036.1 | 16s | GCGAAAGCTTTGCTAATAC | GGCTTGGTAAGCTTTTATC | This study |
| Aerococcus | JQ962617.2 | 16s | TTCGGATCGTAAAACTCTG | CGTGGCTTTCTGGTAAGA | This study |
| *Atopobium vaginae* | JQ511973.1 | 16s | GTTAGGTCAGGAGTTAAATCTG | TCATGGCCCAGAAGACC | [[1](#_ENREF_1)] |
| BVAB1 | AY724739.1 | 16s | AGTGTAGGCGGCACTATAAG | AGCCTTAGCGTCAGTTATCG | [[1](#_ENREF_1)] |
| BVAB2 | AY724740.1 | 16s | AGGCGGCTAGATAAGTGTGA | TCCTCTCCAGCACTCAAGCTAA | [[1](#_ENREF_1)] |
| BVAB3 | AY724741 | 16s | CCCTTGAACGATGTAGAGATACATAA | GCTGCTCTCTGTTGTAGCCATT | [[1](#_ENREF_1)] |
| BVAB-TM7 | AY738690.1 | 16s | AACTGCTTGGCTCGAGATTATC | TCTCCTTTCGGAGAAATTCTAGG | [[2](#_ENREF_2)] |
| Cloacibacterium | JQ962488.2 | 16s | CGGCATCGTTTAATATTGAA | CCCCTAAAAGATCATCGC | This study |
| Corynebacterium | NR_025436.1 | 16s | GGGTAATGGCCTACCAAG | CCGTATCTCAGTCCCAATG | This study |
| *Dialister spp* | JX104025.1 | 16s | CCTAGTGTAGCGGTGAAA | GTCAGTTTTCGTCCAGAA | This study |
| *E. coli* universal | CP002967.1 | beta-D-glucuronidase | GTGTGATATCTACCCGCTTCGC | AGAACGGTTTGTGGTTAATCAGGA | [[3](#_ENREF_3)] |
| Eggerthella spp | AY959023.1 | 16s | GGTTGCTCAAGCGGAACCTCTAAT | AATTCCATCTGCCTGTACCGCACT | [[1](#_ENREF_1)] |
| Eggerthella-like | AY738656.1 | 16s | AACCTCGAGCCGGGTTCC | TCGGCACGGAAGATGTAATCT | [[2](#_ENREF_2)] |
| Enterococcus universal | HF558530.1 | 23s | AGAAATTCCAAACGAACTTG | CAGTGCTCTACCTCCATCATT | [[4](#_ENREF_4)] |
| *Gardnerella vaginalis* 154 | JX860322.1 | 16s | CTCTTGGAAACGGGTGGTAA | TTGCTCCCAATCAAAAGCGGT | [[5](#_ENREF_5)] |
| *G. vaginalis* sialidase | CP002725.1 | sialidase | GACGACGGCGAATGGCACGA | AGTCGCACTCCGCGCAAGTC | [[6](#_ENREF_6)] |
| Haemophilus universal | FQ312002.1 | 16s | GGAGTGGGTTGTACCAGAAGTAGAT | AGGAGGTGATCCAACCGCA | [[7](#_ENREF_7)] |
| hGAPDH | NM_002046.4 | GAPDH | CAACTACATGGTTTACATGTTC | CTCGCTCCTGGAAGATG | [[8](#_ENREF_8)] |
| Lachnospiraceae | X104039.1 | 16s | ATCCGGATTTACTGGGTG | CTCCAAATATCTACGCATTTC | This study |
| Lactobacillus (universal) |  | 16s | TGGAAACAGRTGCTAATACCG | GTCCATTGTGGAAGATTCCC | [[9](#_ENREF_9)] |
| *Lactobacillus crispatus* | JQ805668.1 | 16s | AGCGAGCGGAACTAACAGATTTAC | AGCTGATCATGCGATCTGCTT | [[9](#_ENREF_9)] |
| *Lactobacillus fermentum* | EU621848.1 | 16s | TGGCCCAATTGATTGATG | GCATCTGTTTCCAAATGTTG | This study |
| *Lactobacillus gasseri* | JQ413225.1 | 16s | AGCGAGCTTGCCTAGATGAATTTG | TCTTTTAAACTCTAGACATGCGTC | [[10](#_ENREF_10)] |
| *Lactobacillus iners* | NR_036982.1 | 16s | ACAGGGGTAGTAACTGACCTTTG | ATCTAATCTCTTAGACTGGCTATG | [[2](#_ENREF_2)] |
| *Lactobacillus jensenii* | AB289171.1 | 16s | GCCTATAGAAATTCTTCGGAATGGACA | CAAATGGTATCCCAGACTTAAGGG | [[1](#_ENREF_1)] |
| *Lactobacillus reuteri* | JQ897938.1 | 16s-23s inter. spacer | CAGACAATCTTTGATTGTTTAG | GCTTGTTGGTTTGGGCTCTTC | [[11](#_ENREF_11)] |
| *Lactobacillus rhamnosus* | JX556102.1 | 16s | TGCTTGCATCTTGATTTAATTTTG | GGTTCTTGGATYTATGCGGTATTAG | [[9](#_ENREF_9)] |
| *Leptotrichia amnionii* | JQ839147.1 | 16s | GAGGAAGTTTAGCTTGCTAAATGGAC | CTTTAGTGCCGTAGCTTTCATTTGC | [[1](#_ENREF_1)] |
| *Megasphaera sp type 1* | EF120358.1 | 16s | GACGGATGCCAACAGTATCCGTCCG | AAGTTCGACAGTTTCCGTCCCCTC | [[1](#_ENREF_1)] |
| *Megasphaera sp type 2* | AY738697.1 | 16s | AAGGTGGTAAATAGCCATCATGAG | CTCTCCGACACTCAAGTCTTC | [[2](#_ENREF_2)] |
| *Mobiluncus curtisii* | CP001992.1 | 16s | GCGATGGTTCCAGAGATGGGCCAGCCTT | CACGAGTCCCCGGCCGAA | [[1](#_ENREF_1)] |
| *Mobiluncus mulieris* | X82602.1 | 16s | GCGACATGCCAGAGATGGTGTG | CACGAGTCCCCACCATAACGTG | [[1](#_ENREF_1)] |
| *Parvimonas spp* | JQ259702.1 | 16s | GTCTGAGAGGATGAACGG | CTCCTATGATACCGTCATTATC | This study |
| *Peptoniphilus lacrimalis* | AY738692.1 | 16s | AAGAGACGAACTTAGAGATAAGTTTT | CACCTTCCTCCGATTTATCATC | [[2](#_ENREF_2)] |
| *Peptoniphilus spp* | AY738691.1 | 16s | GACCGGTATAGAGATATACCCT | CACCTTCCTCCGATTTATCATC | [[2](#_ENREF_2)] |
| *Peptostreptococcus spp* | EF120365.1 | 16s | TCATAGGAGGAAGCCCTGGCTAAA | TAAGCTCCACGCTTTGACACCTGA | [[1](#_ENREF_1)] |
| *Prevotella buccalis*-like | JN867243.1 | 16s | GTGCATTGCAGGTAGCGCATGAAT | TACCGTGCACTCAAGCCAAACAGT | [[1](#_ENREF_1)] |
| Prevotella G1 | AY738677.1 | 16s | GTCCCTTATTGCATGTACCATAC | GCCGCTAACACTAGGTGCTA | [[2](#_ENREF_2)] |
| *Prevotella spp* | JN867318.1 | 16s | GGGATGCGTCTGATTAGCTTGTT | CTGCACGCTACTTGGCTGGTTC | [[1](#_ENREF_1)] |
| Proteobacteria α, β |  | rpoB | GGGCAGCGTTTCGGCGAAATGGA | GTCCGACTTGACGGTCAACATTTCCTG | [[12](#_ENREF_12)] |
| Proteobacteria β, γ and Pseudomonadaceae |  | rpoB | CAGGAGTCGTTCAACTCGATCTACATGAT | ACGCCATCAGGCCACGCAT | [[12](#_ENREF_12)] |
| Ruminococcaceae | JX543432.1 | 16s | CAACGCGAAGAACCTTAC | ACGGCAGTCCTATTAGAG | This study |
| *Sneathia sanguinegens* | EF120363.1 | 16s | GATGGGAGCTAGCTTGCTAGAAGAAG | GCTCTCATATAGCGTATTGCTACC | [[1](#_ENREF_1)] |
| Staphylococcus universal |  | elongation factor Tu | GAACGTGGTCAAATCAAAGTTGGTGAAGA | GTCACCAGCTTCAGCGTAGTCTAATAA | [[13](#_ENREF_13)] |
| Streptococcus group B | CP003810.1 | cAMP factor | TTTCACCAGCTGTATTAGAAGTA | GTTCCCTGAACATTATCTTTGAT | [[14](#_ENREF_14)] |
| Streptococcus universal |  | 16s | AGTCGGTGAGGTAACCGTAAG | AGGAGGTGATCCAACCGCA | [[7](#_ENREF_7)] |
| Universal 16s |  | 16s | TCCTACGGGAGGCAGCAGT | GGACTACCAGGGTATCTAATCCTGTT | [[15](#_ENREF_15)] |
| *Veillonella spp* | JQ680348.1 | 16s | GACGAAAGTCTGACGGAG | CCGATTAACAGAGCTTTACAA | This study |

**Table S1. PCR target data for the analysis of the VMB.**

1. Zozaya-Hinchliffe M, Lillis R, Martin DH, Ferris MJ (2010) Quantitative PCR assessments of bacterial species in women with and without bacterial vaginosis. J Clin Microbiol 48: 1812-1819.

2. Fredricks DN, Fiedler TL, Thomas KK, Oakley BB, Marrazzo JM (2007) Targeted PCR for detection of vaginal bacteria associated with bacterial vaginosis. J Clin Microbiol 45: 3270-3276.

3. Frahm E, Obst U (2003) Application of the fluorogenic probe technique (TaqMan PCR) to the detection of Enterococcus spp. and Escherichia coli in water samples. J Microbiol Methods 52: 123-131.

4. Haugland RA, Siefring SC, Wymer LJ, Brenner KP, Dufour AP (2005) Comparison of Enterococcus measurements in freshwater at two recreational beaches by quantitative polymerase chain reaction and membrane filter culture analysis. Water Res 39: 559-568.

5. Henriques A, Cereija T, Machado A, Cerca N (2012) In silico vs in vitro analysis of primer specificity for the detection of Gardnerella vaginalis, Atopobium vaginae and Lactobacillus spp. BMC Res Notes 5: 637.

6. Santiago GL, Deschaght P, El Aila N, Kiama TN, Verstraelen H, et al. (2011) Gardnerella vaginalis comprises three distinct genotypes of which only two produce sialidase. Am J Obstet Gynecol 204: 450.e451-457.

7. Matar GM, Sidani N, Fayad M, Hadi U (1998) Two-step PCR-based assay for identification of bacterial etiology of otitis media with effusion in infected Lebanese children. J Clin Microbiol 36: 1185-1188.

8. Bourne N, Pyles RB, Yi M, Veselenak RL, Davis MM, et al. (2005) Screening for hepatitis C virus antiviral activity with a cell-based secreted alkaline phosphatase reporter replicon system. Antiviral Res 67: 76-82.

9. Byun R, Nadkarni MA, Chhour KL, Martin FE, Jacques NA, et al. (2004) Quantitative analysis of diverse Lactobacillus species present in advanced dental caries. J Clin Microbiol 42: 3128-3136.

10. Tamrakar R, Yamada T, Furuta I, Cho K, Morikawa M, et al. (2007) Association between Lactobacillus species and bacterial vaginosis-related bacteria, and bacterial vaginosis scores in pregnant Japanese women. BMC Infect Dis 7: 128.

11. Song Y, Kato N, Liu C, Matsumiya Y, Kato H, et al. (2000) Rapid identification of 11 human intestinal Lactobacillus species by multiplex PCR assays using group- and species-specific primers derived from the 16S-23S rRNA intergenic spacer region and its flanking 23S rRNA. FEMS Microbiol Lett 187: 167-173.

12. Ecker DJ, Sampath R, Blyn LB, Eshoo MW, Ivy C, et al. (2005) Rapid identification and strain-typing of respiratory pathogens for epidemic surveillance. Proc Natl Acad Sci U S A 102: 8012-8017.

13. Wolk DM, Blyn LB, Hall TA, Sampath R, Ranken R, et al. (2009) Pathogen profiling: rapid molecular characterization of Staphylococcus aureus by PCR/electrospray ionization-mass spectrometry and correlation with phenotype. J Clin Microbiol 47: 3129-3137.

14. Ke D, Ménard C, Picard FJ, Boissinot M, Ouellette M, et al. (2000) Development of conventional and real-time PCR assays for the rapid detection of group B streptococci. Clin Chem 46: 324-331.

15. Mohammadi T, Reesink HW, Vandenbroucke-Grauls CM, Savelkoul PH (2003) Optimization of real-time PCR assay for rapid and sensitive detection of eubacterial 16S ribosomal DNA in platelet concentrates. J Clin Microbiol 41: 4796-4798.
